# Innovative Poly (Vinylidene Fluoride) (PVDF) Electrospun Nanofiber Membrane Preparation Using DMSO as a Low Toxicity Solvent

**DOI:** 10.3390/membranes10030036

**Published:** 2020-02-26

**Authors:** Francesca Russo, Claudia Ursino, Elisa Avruscio, Giovanni Desiderio, Andrea Perrone, Sergio Santoro, Francesco Galiano, Alberto Figoli

**Affiliations:** 1 Institute on Membrane Technology (ITM-CNR), Via Pietro Bucci, Cubo 17/C, 87036 Rende (CS), Italy; f.russo@itm.cnr.it (F.R.); c.ursino@itm.cnr.it (C.U.); elisa.avruscio@gmail.com (E.A.); f.galiano@itm.cnr.it (F.G.); 2 Laboratory of Industrial and Synthetic Organic Chemistry (LISOC), Department of Chemistry and Chemical Technology, University of Calabria, via P. Bucci 12/C, 87036 Rende (CS), Italy; 3 CNR/Nanotec c/o Dipartimento di Fisica, Università della Calabria, 87036 Rende (CS), Italy; giovanni.desiderio@fis.unical.it; 4 DeltaE srl, C/o Universitá della Calabria, Via Pietro Bucci cubo 31D, Arcavacata di Rende, 87036 Rende (CS), Italy; 5 Department of Environmental Engineering (DIAm), Università della Calabria, Via Pietro Bucci, Cubo 44/A, 87036 Rende (CS), Italy; sergio.santoro@unical.it

**Keywords:** DMSO, low toxic solvent, electrospinning, electrospun fiber membranes (ENMs), water treatment, membrane preparation

## Abstract

Electrospinning is an emerging technique for the preparation of electrospun fiber membranes (ENMs), and a very promising one on the basis of the high-yield and the scalability of the process according to a process intensification strategy. Most of the research reported in the literature has been focused on the preparation of poly (vinylidene fluoride) (PVDF) ENMs by using N,N- dimethylformamide (DMF) as a solvent, which is considered a mutagenic and cancerogenic substance. Hence, the possibility of using alternative solvents represents an interesting approach to investigate. In this work, we explored the use of dimethyl sulfoxide (DMSO) as a low toxicity solvent in a mixture with acetone for the preparation of PVDF-ENMs. As a first step, a solubility study of the polymer, PVDF 6012 Solef^®^, in several DMSO/acetone mixtures was carried out, and then, different operating conditions (e.g., applied voltage and needle to collector plate distance) for the successful electrospinning of the ENMs were evaluated. The study provided evidence of the crucial role of solution conductivity in the electrospinning phase and the thermal post-treatment. The prepared ENMs were characterized by evaluating the morphology (by SEM), pore-size, porosity, surface properties, and performance in terms of water permeability. The obtained results showed the possibility of producing ENMs in a more sustainable way, with a pore size in the range of 0.2–0.8 µm, high porosity (above 80%), and water flux in the range of 11.000–38.000 L/m^2^·h·bar.

## 1. Introduction

Membrane technology plays a key role in all those processes where water treatment is required, e.g., desalination and wastewater treatment. Membrane processes, in fact, include several separation techniques, such as microfiltration (MF), ultrafiltration (UF), nanofiltration (NF), reverse osmosis (RO), membrane distillation (MD), pervaporation (PV), etc. Membrane processes are widely recognized for their numerous advantages, including low energy consumption, relatively small footprint, mild operating conditions, and environmentally friendly approaches [1,2]. Depending on the polymeric material, but also on the application, porous membranes can be prepared according to several techniques- Some of which are already well consolidated and diffused, such as phase inversion. Others are more innovative and still at an embryonic stage, such as electrospinning. In fact, only in recent years has the electrospinning technique been gaining popularity for the preparation of polymeric nanofibrous membranes, which are appreciated for their undoubtable advantages such as high level of versatility, vast material selection, and one step preparation [3]. Electrospun nanofiber membranes (ENMs) exhibit several properties that make them suitable for several applications in water treatment processes, thanks to their high specific surface area and porosity, and large number of inter-/intra fibrous pores [3,4]. The electrospinning technique uses electrostatic forces to produce fine fibers (from nanometer to micrometer) from polymer solutions or melts. The system is based on three major components: a high voltage power supply, a spinneret, and a collector [4,5]. The parameters influencing the final ENMs are various and include the electrospinning system (electric field, flow rate, distance between needle and collector), humidity, temperature, and polymeric dope solution in terms of viscosity, solution conductivity, polymer concentration, and solvent. In this regard, most of the solvents currently employed in electrospinning are toxic and harmful. During the electrospinning process, the continuous evaporation of the solvent leads to a significant accumulation of its vapors into the surrounding environment, which poses serious concerns about human health safeguards and environmental pollution [6]. In a typical electrospinning process, in order to get a homogeneous spinning solution, polymers are dissolved in high polar organic solvents, such as tetrahydrofuran (TFH), N-Methyl-2-pyrrolidone (NMP), N,N-dimethylformamide (DMF), or dimethylacetamide (DMA) [6,7,8,9]. Despite the large use of these solvents in different fields [10], because of their toxicity and harmfulness, the European Chemicals Agency included them in the “Candidate list of substances of very high concern for authorization” [11,12]. Today, more than ever, there is increasing attention and awareness on environmental and health problems [13] and the possibility of employing “green” solvents, as alternatives to traditional ones, is considered as a valid route that can be pursued for minimizing the impact of hazardous substances [14]. According to this scenario, in this research, poly (vinylidene fluoride) (PVDF) electrospun fiber membranes (ENMs) were prepared using dimethyl sulfoxide (DMSO) as an alternative low toxicity solvent in a mixture with acetone, which was used as a co-solvent [12,14,15]. DMSO has a low toxicity and not hazardous solvent [16] able to solubilize high concentrations of PVDF at room or mild temperatures [14]. The use of DMSO for membrane preparation has already been largely documented in the literature. Marino et al [12], for instance, prepared polyethersulfone (PES) MF membranes via phase separation using a pleasant-smelling version of DMSO named DMSO EVOL^TM^. The preparation of UF and NF PES membranes using DMSO as a low toxicity solvent was also reported by Evenepoel et al [15]. A predictive study of membrane morphology via a ternary phase diagram for a water/DMSO/PES system was performed by Madaeni et al [17]. The use of DMSO as an alternative solvent was also studied for preparation of PVDF membranes via phase separation [18,19,20]. Thermodynamic and kinetic studies and the influence of different variables on PVDF membranes’ physicochemical properties have been reported by Enayatzadeh et al [21] and Arefi-Oskoui et al [22]. DMSO was employed as a solvent for the preparation of ENMs such as in PVDF, polyacrylonitrile (PAN) [23,24], and poly(vinyl) alcohol (PVA) [25,26]. The influence of the solvents’ properties, including DMSO, for polyurethane (PU) nanofibers was also reported by Mondal [27], and more recently an interesting study of a series of new polymers (poly(vinyl alcohol) (PVOH), poly(2ethyl2oxazolene) (PEOZ), poly(vinylpyrrolidone) (PVP), poly(styrene-co-acrylonitrile) (SAN), poly(vinyl alcohol-co-ethylene) (EVOH), and acrylonitrile butadiene styrene (ABS), which are applicable for electrospinning from DMSO, was reported by Wortmann et al [28]. In the case of PVDF, the use of toxic solvents for ENM preparation has most often been reported [29,30,31,32,33,34]. 

In this work, PVDF ENMs with pore sizes in the microfiltration (MF) range were successfully prepared using DMSO as a solvent and compared to ENMs prepared with DMF.

Several parameters were investigated, such as polymer concentration (6-8-10 wt.%), the presence of LiCl as additive in the dope solution, electrospinning parameters (voltage and needle distance to collector), and the membranes post-treatment type. The PVDF ENMs were characterized in terms of morphology (by scanning electron microscopy and atomic force microscopy, contact angle, porosity, pore size, and pure water permeability. 

## 2. Materials and Methods

### 2.1. Materials 

The polymer, PVDF 6012 Solef^®^, was supplied by Solvay Specialty Polymers (Bollate, Italy). DMSO DMF, isopropanol, LiCl, and acetone were purchased from Sigma Aldrich (Milan, Italy). The polymer was desiccated under vacuum at 40 °C for 6 h before use. Distillate water was used for the post-treatment.

### 2.2. Dope Solutions Preparation

Dope solutions were obtained by dissolving the powder of the PVDF polymer in the mixture of solvents (DMSO/acetone 6:4) with and without LiCl (0.43 wt%) at 70 °C. After 30 min, a homogeneous solution was obtained, and it was degassed at room temperature (25 °C) for 2 h. In order to determine the optimal viscosity of the solution, different concentrations of polymer were studied (6, 8, 10 wt%). The same protocol was employed for the preparation of the PVDF solution using DMF as a solvent. 

### 2.3. ENMs Preparation by Electrospinning 

**Figure 1:**
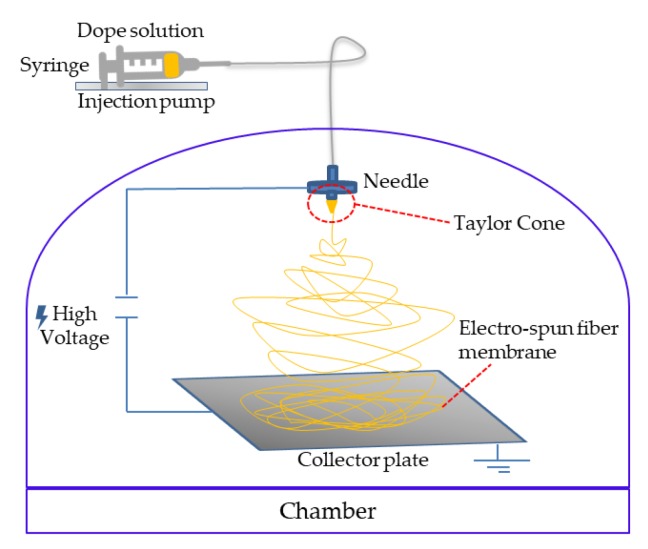
Schematic representation of the electrospinning set-up used in this study.

The stable solution at room temperature was transferred in a syringe and fed to the electrospinning needle. The electrospinning machine used in this study was supplied by DeltaE srl and is depicted in Figure 1. 

The fibers were prepared at the optimal solution flow rate of 1 mL h^−1^. At higher flow rates, the effect of the gravitational force was noticeable and the electric field was unable to draw the whole polymeric jet, thus, droplets were observed as result. The rate of deposition of the ENMs is directly proportional to the flow rate of the polymeric solution. Five hours were required to produce 345 cm^2^ of ENMs. The effect of the electric field on the ENMs was evaluated by varying the difference of voltage between the needle and the plate (12, 15 and 18 kV). The distance between the needle and the collector plate was varied between 10 and 20 cm. In order to obtain a homogeneous thickness of the ENMs, the needle was moved in 2 dimensions (left-right and front-rear). After the deposition of the ENMs, two membrane post-treatment were studied: (1) at 100 °C in the oven for 1 h and then overnight at 130 °C between two glass plates and (2) exposed to air for 2 h, followed by immersion in a water bath for 24 h and then overnight at 40 °C in an oven. In Table 1, the ENM prepared in this study and the investigated parameters are reported. 

### 2.4. ENM Characterizations

#### 2.4.1. Scanning Electron Microscopy (SEM)

Scanning electron microscopy (SEM) (Zeiss-EVO MA10 instrument, Milan, Italy) was employed to study the morphology of the prepared ENMs. Samples were coated with a thin layer of gold (sputter machine Quorum Q 150R S) prior to analyses in order to make them conductive.

#### 2.4.2. Porosity

Porosity was determined using the following Equation (1):

(1) $$\mathrm{Porosity}\;(\%)=\frac{\frac{wt_{w}-wt_{d}}{\rho_{k}}}{\frac{wt_{w}-wt_{d}}{\rho_{k}}+\frac{wt_{d}}{\rho_{p}}}\;\cdot 100$$

where wt_w_ is the weight of the membrane in the wet state, wt_d_ is the weight of the membrane in the dry state, ρ_k_ is the isopropanol density, and ρ_p_ is the polymer density. Each value of porosity is the average of three different measurements.

#### 2.4.3. Pore Size

A capillary flow porometer instrument- CFP-1500 AEXL (Porous Materials Inc., Ithaca, NY, USA) was used for the measurement of the mean flow pore diameter and pore distribution through a liquid-gas process. Porewick^®^ (surface tension of 16 dyne/cm) was used as a wetting liquid.

#### 2.4.4. Contact Angle 

CAM200 Instrument, Nordtest srl, GI, Serravalle Scrivia (AL) Italy, was employed for the evaluation of the membrane contact angle by using the sessile drop method. For each membrane, the average and standard deviation of five static measurements was calculated. 

#### 2.4.5. Thickness 

Membrane thickness was evaluated by a digital micrometer (precision of ±0.001 mm) from Carl Mahr (Germany). The average value was assessed on five regions for each membrane.

#### 2.4.6. Atomic Force Microscopy (AFM)

The measurements of surface roughness were determined by the AFM by means of a Bruker Multimode 8 with Nanoscope V Controller.

#### 2.4.7. Water Permeability (PWP) 

A cross-flow cell with an area of 8 cm^2^ (DeltaE srl, Italy) was used to determine the permeability of the ENMs. Pure water was forced to pass across the membrane by means of a gear pump (Tuthill Pump Co., California). The values of permeability were measured at the steady state condition (after an equilibrium of 30 min at 1 bar). The PWP was calculated using Equation (2):

(2) $$\mathrm{PWP}=\frac{Q}{A\cdot t\cdot p}$$

where A is the membrane area (m^2^), p is the pressure (bar), Q is the permeate volume (L), and t is the time (h).

## 3. Results

### 3.1. Viscosity Meaurement

In Table 2, the viscosity values of the investigated dope solutions are reported. Viscosity represents a crucial parameter to ensure the success of the ENMs’ preparation. DS1, prepared using the DMF/acetone solvent mixture, showed a viscosity of about 87 cP. This value is in agreement with what has already been reported in the literature and considered ideal for the electrospinning of PVDF nanofibers [35]. In order to get similar values with the new solvent DMSO, four solutions (DS2-DS5) with different polymer concentrations (6, 8, and 10 wt%) were considered. DS2 and DS4 solutions, prepared with 8 wt% of PVDF and differing for the presence of LiCl, showed viscosity values of 79.4 and 74.4 cP and were closer to the DS1 solution used as a reference. 

The dope solutions DS3 (6 wt% of PVDF) and DS5 (10 wt% of PVDF) presented a viscosity of 49.6 and 199.3 cP, respectively. DS5’s viscosity was too high and not suitable for electrospinning the nanofibers, due to the difficulty of feeding the dope solution into the needle without causing it to clog. The viscosity of DS3, on the contrary, was too diluted for the formation of a continuous jet during the elettrospinning process and was responsible for the formation of defects in the nascent nanofibers [36].

### 3.2. Morphology of ENMs

**Figure 2:**
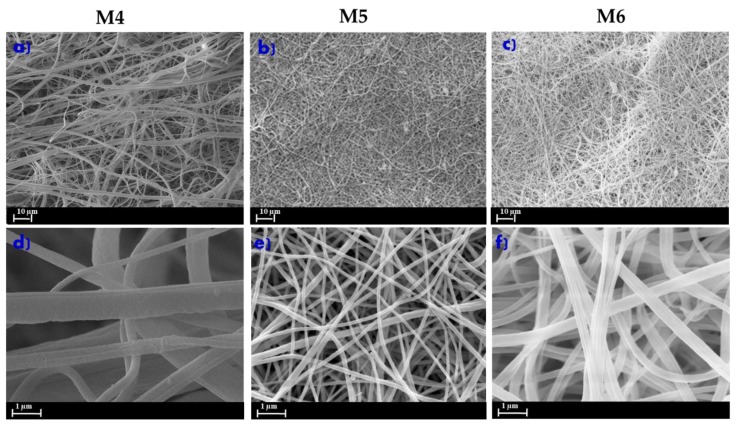
SEM pictures of the electrospun fiber membranes (ENMs) at different poly (vinylidene fluoride) (PVDF) polymer concentrations: (**a**) M4 (6 wt% PVDF), (**b**) M5 (8 wt% PVDF), (**c**) M6 (10 wt% PVDF) at 10 µm; (**d**) M4 (6 wt% PVDF), (**e**) M5 (8 wt% PVDF), (**f**) M6 (10 wt% PVDF) at 1 µm. Conditions of electrospinning process: 15 kV voltage and 15 cm of needle to collector distance.

Figure 2 shows the morphology of the ENMs at different polymer concentrations and at the same electrospinning conditions (15 kV of voltage and 15 cm of needle to collector distance) in the presence of LiCl. All the developed PVDF-ENMs showed a randomly-oriented fibrous morphology. The polymer concentration played an important role in the morphology of the nanofibers, which is strictly related to the polymer solution viscosity [37]. In fact, at low polymer concentration, such as the case of M4 (Figure 2a,d), the nanofibers networks were heterogeneous and the formation of beads and non-uniform fibers was observed. A solution with low PVDF concentration implies a minimal viscoelastic force unable to match the electrostatic and columbic forces that stretch the jet [38]. The consequences are the partial break of the jet (heterogenous fibers) and formation of spherical beads under the effects of surface tension [38]. Viscoelastic forces increase by increasing the PVDF content, thus, the partial breakup of the electrospun jet is prevented and polymer entanglement is optimized, enabling the solvent molecules to be distributed over the entangled chains, favoring the formation of smooth and homogenous fibers [39]. With the increase of polymer concentration (M5), in fact, a more regular structure, characterized by ultrafine and straight nanofibers (Figure 2b,e), was observed. The average diameter for M5 fibers was around 293 nm. By increasing the polymer concentration from 8 wt% to 10 wt% (M6), the ENMs’ morphology was more irregular and thicker, with a diameter ranging from 321 to 623 nm (Figure 2f). This effect was mainly due to the high solution viscosity responsible for the entanglement of the polymer chain macromolecules [36].

**Figure 3:**
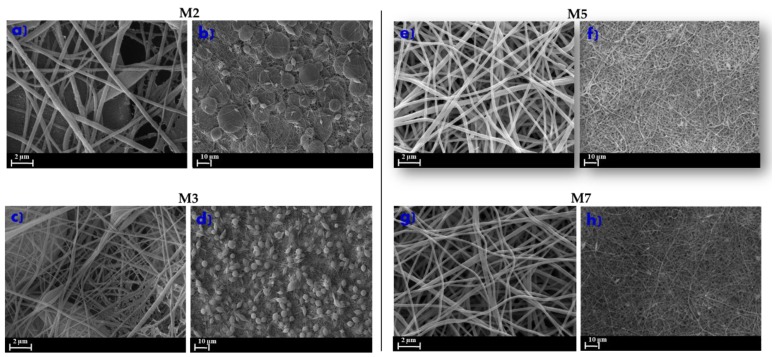
SEM pictures of the ENMs using dimethyl sulfoxide (DMSO)/acetone as a mixture of solvents at different post-treatments with and without LiCl. M2 (**a**) and (**b**); M3 (**c**) and (**d**); M5 (**e**) and (**f**); M7 g and h. Conditions of electrospinning process: 15 kV voltage and 15 cm of needle to collector distance.

The effect of salt additions on the electrospinnability of ENMs is largely documented in the literature [5,40,41]. The presence of a salt (such as LiCl) plays a crucial role in achieving a good balance in terms of conductivity, viscosity, and surface tension, allowing one to obtain homogeneous nanofibers. Figure 3a–d shows the morphology of ENMs prepared without and with LiCl. As can be observed, significant beads formations (grain-like or branched structures) characterized the morphology of M2 and M3 membranes (Figure 3a–d) prepared without LiCl. 

The addition of LiCl (membranes M5 and M7 in Figure 3e–h), on the contrary, considerably improved the quality of the nanofibers by increasing the electrical conductivity of the polymer solution, which resulted in a higher charge density on the surface of the charged jet. This fostered the stretching of the solution jet as a consequence of higher level of charges, facilitating the electrospinning process.

In Figure 3, the influence of post-treatment on the ENMs’ morphology is reported. 

Although, in our case, no relevant effects were observed on the morphology of the polymeric network submitted to different post-treatments, this step is generally important for producing nanofibers with a high level of integrity and cohesion. Moreover, it is crucial for the formation of interconnected pores and in defining the surface roughness. Liao et al. [29] examined the properties of PVDF nanofibers with LiCl pressed between two glass plates at 170 °C for 1 h. Electrospun membranes displaying uniform diameter with a pore size in the range of 0.50–0.33 µm were produced. Shirazi et al. [42] found that heat treatment up to 150 °C contributed to the formation of more uniform and smaller pores. Santoro et al. [34,35] used different temperatures for thermal treatment: 100 °C for 1 h and then 130 °C between two glass plates were essential for getting consistent nanofibers. 

In this work, two different post-treatments were investigated:

1. 100 °C in the oven for 1 h and then overnight at 130 °C between two glass plates - membranes M3 (Figure 3c,d) and M7 (Figure 3g,h). This approach was based following information in the literature [35].
2. Exposure to air for 2 h, followed by immersion in a water bath for 24 h and then overnight at 40 °C—membranes M2 (Figure 3a,b) and M5 (Figure 3e–f). This approach was first proposed in this study.

The post treatment proposed in the present work (membranes M2 and M5) allows the removal of LiCl and of solvent traces, thanks to the immersion of the prepared ENMs in the water bath. Moreover, it requires significantly lower temperatures in comparison to the method proposed by Santoro et al. [35] without compromising the ENMs’ properties. 

**Figure 4:**
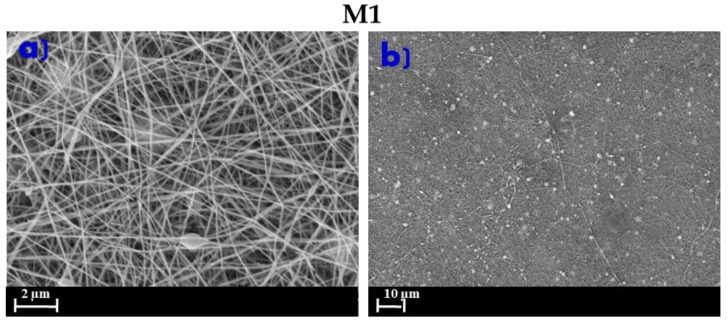
SEM pictures of ENMs prepared using N,N- dimethylformamide (DMF)/acetone as a mixture of solvents. (**a**) M1 2 µm; (**b**) M1 10 µm. Conditions of electrospinning process: 15 kV voltage and 15 cm of needle to collector distance.

Figure 4a,b shows the morphology of the M1 membrane prepared using DMF as a solvent and used as a comparison.

The M1 morphology was characterized by a 3D network of fibers with a small number of nanobeads, probably due to the interaction of the solvent with the salt (LiCl) [43]. As reported in the literature, LiCl strongly interacts with DMF solvent to form stable solutions that decrease the strength of the solvent [44,45,46]. The high viscosity of the polymer dope solution with DMF and LiCl (DS1) suggested that this interaction was not sufficient to produce totality uniform nanofibers.

In the electrospinning process, the effect of the solvent is crucial for the preparation of uniform ENMs. An ideal solvent should favor the stability of the solution at low polymer concentrations for obtaining uniform 3D networks of the nanofiber by reducing the overall energy correlated to the orientation of polymer chains in the solution. In this regard, by comparing M1 and M5 membranes (prepared under the same conditions but with DMF and DMSO as solvents, respectively), the latter one showed a better distribution and uniformity of the fibers. 

DMSO is, in fact, considered a good solvent for PVDF, as can be deduced by the Hansen solubility parameters (HSPs). Table 3 summarizes the HSPs for PVDF, DMSO, and DMF [47] considering these three components: the dispersion forces component (δ_d_), the polar force component (δ_p_), and the hydrogen bonding component (δ_h_), as reported in Table 3. 

The values in Table 3 predict well the solubility of the polymer for both solvents based on the concept of “like dissolves like” [47], as indicated by the similar values of total solubility (ẟ_T_). 

The dielectric constant of the solvent can also influence the spinnability of a polymer solution. For some polymers, in fact, a higher dielectric constant (such as the one exhibited by DMSO) can enhance the electrospinnability, decreasing the formation of beads [48,50]. 

**Figure 5:**
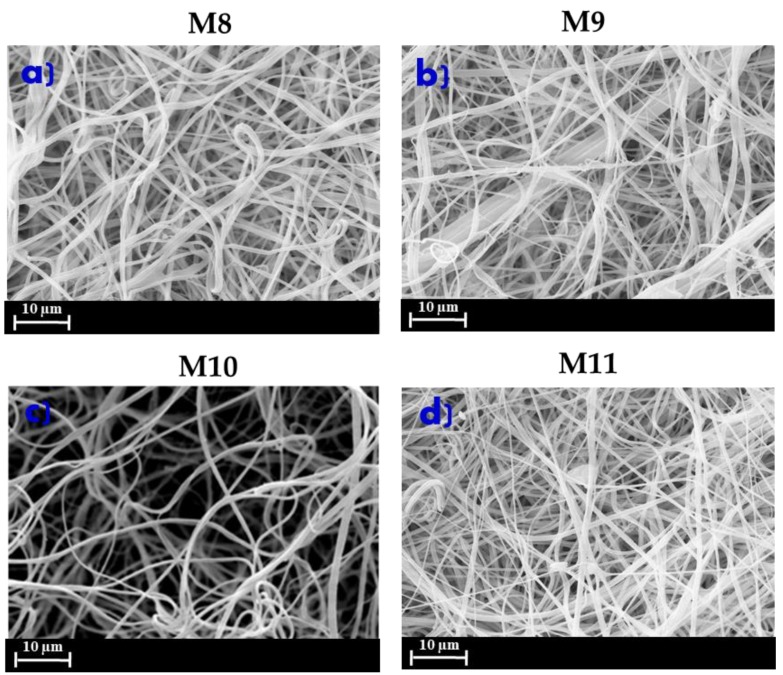
SEM pictures of ENMs using DMSO/acetone as a mixture of solvents at different voltages ((**a**) and (**b**): M8 and M9 membranes) and needle to collector distance ((**c**) and (**d**): M10 and M11 membranes).

However, the effect of both conditioning processes on membrane morphology, was investigated. Figure 5a,b shows the morphology of PVDF ENMs prepared at different voltages of 12 (M8) and 18 kV (M9), while Figure 5b,c shows the morphology of PVDF ENMs prepared at different needle to collector distances of 10 cm (M10) and 20 cm (M11). It should be noted that in all cases, there was an absence of beads or defects, which are usually observed in ENMs prepared by employing DMF as a solvent [34,35,36].

The electrical field is the driving force of the jet initiation and fiber stretching. Voltage values below or higher than 15kV resulted in ENMs characterized by heterogeneous and non-uniform fibers (Figure 5a,b). 

The needle-collector distance affects the stretching and the deposition of ENMs. A higher distance provides a longer time for solvent evaporation and polymer precipitation, and space for fiber stretching. This explains the formation of coarse fibers in the case of M10 (Figure 5c) due to the insufficient distance between the needle and the collector in comparison to M11 (Figure 5d). 

### 3.3. Porosity, Pore Size, Thickness, Contact Angle, and AFM of ENMs

In Table 4, the results of the prepared ENMs in terms of contact angle, porosity, pore size, and thickness are reported. 

**Figure 6:**
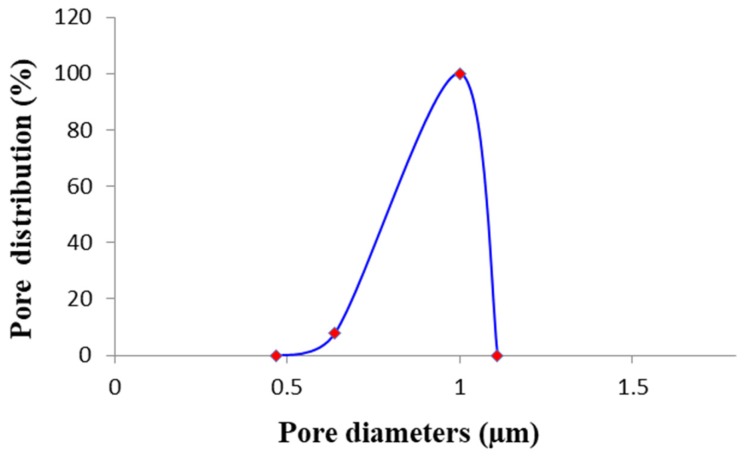
Pore distribution of the M5 ENM.

Generally, the electrospinning process produces nanofiber membranes with high porosity and uniform pore size with interconnected pores [51]. With respect to PVDF flat membranes fabricated by phase inversion (porosity around 80%) [51,52], in fact, the porosity of the developed ENMs membranes was higher than 90%, except for the membrane M2 (porosity of 74% ± 1%). Regarding the pore size, it was found that by increasing the polymer concentration (from M4 to M6) the pore size also increased (from 0.87 µm to 1.03 µm). This can be attributed to the fact that the jet elongation become more difficult and slow at high polymer concentrations [3]. The post-treatment can also the effect membrane pore size [53]. ENMs prepared at 8 wt% of PVDF using the post-treatment with water and 40 °C in the oven (M5) presented an average pore size of 0.9 ± 0.08 µm and a pore size distribution that was very narrow (as reported in the Figure 6), while the same composition of ENMs, but prepared with a post-thermal treatment (100 °C for 1 h and overnight at 130 °C), presented a larger pore size of 1.27 ± 0.02 µm. A significant difference in terms of pore size was observed for the membranes prepared without (M2 and M3) and with LiCl (M5). In particular, the mean pore size decreased drastically from 2.29 µm for M2 and 2.26 µm for M3 to 0.9 µm for M5. This was due to the decrease of surface tension as a consequence of the LiCl addition in the polymer solution. A reduced surface tension, in fact, makes jets elongate easily, allowing the formation a more homogenous network of nanofibers [54]. The change in the applied voltage did not have a big impact on membrane pore size, which was nearly constant and about 1μm (membrane M8 and M9). Also, the distance between the needle and the collector plate (M10 and M11) did not greatly influence the membrane pore size. 

The pore size of M1 prepared with DMF was 0.81 µm, which was similar to membrane M5 prepared with DMSO. 

The contact angle of the prepared ENMs ranged from 114° to 128°, reflecting the hydrohopbic nature of the polymer. 

**Figure 7:**
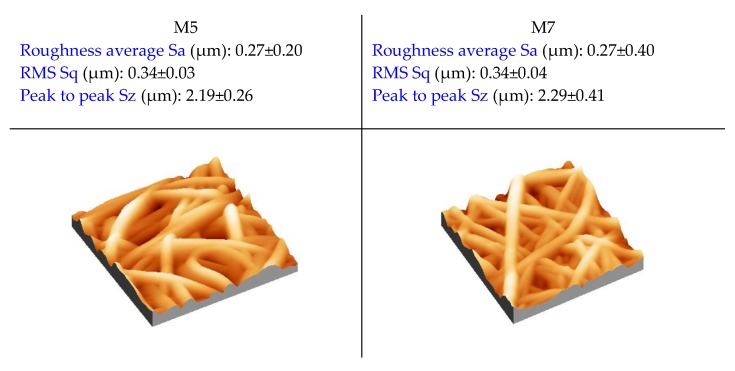
Atomic force microscopy (AFM) pictures of the PVDF ENMs and the values of roughness for M5 and M7.

The roughness was measured for the membranes M5 and M7, which differed from each other in that different post-treatments were applied (Figure 7). Both membranes exhibited the same value of surface roughness (0.27 µm), independent of the post-treatment type adopted. The higher roughness generally measured for electrospun membranes, in comparison to analogous membranes prepared by phase inversion techniques, is generally accompanied by higher contact angle values [19]. This is the reason why in this case, the PVDF ENMs showed higher contact angle values (always above 110°) in comparison to PVDF membranes prepared by phase classic inversion processes (about 90°) [55]. 

The values of thickness for all PVDF ENMs are reported in the Table 4. The thickness increases, as expected, from 65 µm for the membrane M4 to 76 µm for the membrane M6 as the polymer concentration increased (from 6 wt% to 10 wt%). The increase of thickness was also observed in the membrane prepared at high voltage (M9: 79 µm). When the needle to collector plate distance changed from 10 cm (M10) to 20 cm (M11), the thickness increased significantly from 42 µm to 75 µm. The membrane M5 presented a thickness of 73 µm. A similar result was obtained for the membrane M1 prepared with DMF as a solvent (69 µm). Both membranes, in fact, underwent the same post-treatment procedure (dried in air for 2 h and then immersed in water before entering the oven at 40 °C).

On the other hand, the membranes which were subjected to a different post-treatments (between two glass plates), such as M7 and M3, presented the lowest values of thickness (54 and 39 µm, respectively). 

Membranes prepared without LiCl (M2 and M3) presented, in general, lower values of thickness as consequence of a resistance to charge transfer across the surface [56].

**Figure 8:**
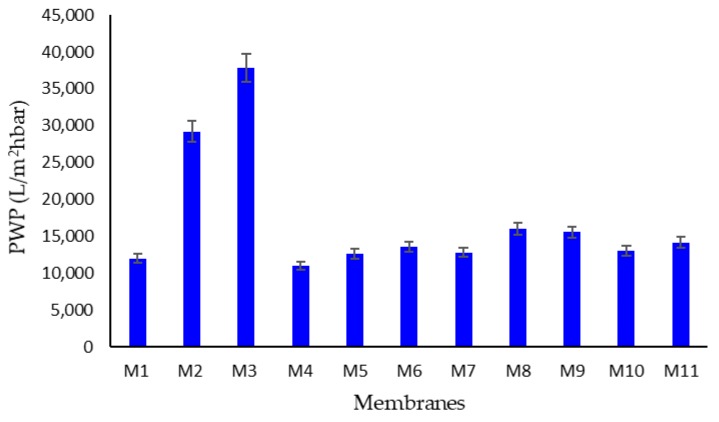
Water permeability (PWP) of prepared ENMs.

During the filtration tests, the ENMs showed excellent permeability with values comparable or superior to the membranes prepared with DMF as a solvent (M1) (Figure 8). The membrane M5 prepared with DMSO as a solvent presented the highest permeability (about 14000 L/m^2^·h·bar), in agreement with its largest pore size (0.90 ± 0.08) µm, with respect to the M1 membrane prepared using DMF as a solvent, at the same conditions (PWP: about 12,500 L/m^2^·h·bar; pore size: 0.81 ± 0.04 µm). The PWP results were higher for the membranes M2 and M3 (29,207 and 37,801 L/m^2^·h·bar, respectively), in alignment with pore size data. By increasing the polymer concentration (from M4 to M6) an increase in PWP (from 11,100 to 13,629 L/m^2^·h·bar) was also observed, and always in agreement with pore size measurement. Slight differences in PWP (about 15,000 L/m^2^·h·bar) were found for ENMs prepared at different voltages (M8 and M9) and at different needle to collector distances (M10 and M11).

## 4. Comparison with the Literature

In order to evaluate the employment of DMSO as a solvent with low toxicity for electrospun membrane preparation, a comparison with the most congruent works on PVDF ENMs is shown in Table 5. As reported, dipolar aprotic solvents, such as DMA, DMF, and NMP were classified as highly harmful, but widely explored for the preparation of PVDF ENMs [11]. The use of less toxic alternatives, such as DMSO, could lay the groundwork for future plans in the suitanible preparation of electrospun membranes. Akduman et al. [57] used a mixture of DMA/acetone with the aim of obtaining nanofibers for the removal of contaminants from wastewater at relatively low cost. Different electrospun membranes were prepared at different polymer concentrations at room temperature, obtaining large pore sizes in the range of 2-5.88 µm. Singh Lalia et al. [54] also studied the effect of polymer concentration on the morphology of electrospun nanofibers, concluding that a decrease of polymer concentration leads to the formation of beads. The nanofibers in this case were prepared with the presence of a continuous hot-press between two layers, obtaining a polymeric network with a pore size of 0.26–1.49 µm. The same procedure was adopted by Na et al. [58], producing heterogeneous PVDF ENMs with large pore sizes. This method is very demanding in terms of energy consumption, and it is not convenient for process intensification. However, several studies have demonstrated that the random distribution of the fibers requires heat treatment to guarantee integrity and mechanical stability to the membranes [29,59]. Lei et al. [59] produced fibers with a high concentration of the polymer and NMP as a solvent, confirming that the morphology was greatly influenced by the volume ratio of the binary solvent system (NMP/acetone). 

Recently, efforts have been focused on the development of PVDF ENMs using DMSO as a low toxicity solvent [60,61]. Saghafi et al. [60] prepared membranes by electrospinning 15 wt% of PVDF solubilized in DMSO/acetone and explored the fracture behavior of carbon/epoxy laminates interleaved with the developed PVDF ENMs. However, the morphological analyses revealed the heterogenity of the PVDF network due to the presence of beads [60]. Gee et al. [61] reported an optimized procedure for the preparation of electrospun membranes (12 wt% of PVDF) using different solvents (DMF, NMP, and DMSO) characterized in terms of piezoelectric properties. The produced membranes presented a pore size larger than 1 µm and the authors concluded that DMF is the most attractive solvent for nanofiber membrane fabrication and filtration performance, suggesting the need of further optimization for the employment of DMSO [61]. This study demonstrated the feasibility of the employment of DMSO for the preparation of homogeneous PVDF ENMs and the opportunity to tune the membrane morphology by varying the operating conditions of the electrospinning properties and/or the composition of the polymeric solution. In fact, this systematic study revealed the opportunity to design PVDF ENMs with high porosity and a sharp pore size, even at value below than 1 μm, which is useful for membrane applications in wastewater treatment. Furthermore, the developed protocol reduced the energy demand of the post-treatment, leading to uniform and self-consistent PVDF ENMs. Thus, this work paves the way for a feasible and sustainable preparation of PVDF ENMs using a low toxicity solvent (DMSO), and their employment in wastewater treatment.

## 5. Conclusions

In this study, a more sustainable route for the production of PVDF ENMs was developed. The ENMs were prepared using DMSO as a low toxicity solvent in a mixture with acetone. Different variables were investigated, such as polymer concentration, the presence of LiCl, the post-treatment type, and the electrospinning parameters (applied voltage and needle to collector plate distance). From the characterization tests performed, the optimal electrospinning process conditions were found to be at 15 kV of applied voltage and 15 cm of needle to collector distance. ENMs with uniform diameter and homogenous distribution were obtained with properties comparable to the analogous ENMs produced with the traditional toxic solvent DMF. The pore size of prepared membranes was in the MF range, suggesting a potential application in water treatment applications. 

## Figures and Tables

**Table 1:** Electrospun fiber membranes (ENMs) produced by electrospinning.

Membrane Code	Solutions					Conditions			
	Polymer	Solvents			Additive	Electrospinning Parameters		Post-Treatment	
	PVDF 6012	DMSO	DMF	Acetone	LiCl	Needle-to-Collector Distance	Voltage	Air for 2 h; Water for 24 h and in the Oven at 40 °C	100 °C in the Oven for 1 h; 130 °C between Two Glass Plates
	(wt%)	(v/v)	(v/v)	(v/v)	(wt%)	(cm)	(kV)		
M1	8	-	6	4	0.43	15	15	X	-
M2	8	6	-	4	-	15	15	X	-
M3	8	6	-	4	-	15	15	-	X
M4	6	6	-	4	0.43	15	15	X	-
M5	8	6	-	4	0.43	15	15	X	-
M6	10	6	-	4	0.43	15	15	X	-
M7	8	6	-	4	0.43	15	15	-	X
M8	8	6	-	4	0.43	15	12	X	-
M9	8	6	-	4	0.43	15	18	X	-
M10	8	6	-	4	0.43	10	15	X	-
M11	8	6	-	4	0.43	20	15	X	-

**Table 2:** Viscosity of the dope solutions prepared in this study.

Dope Solutions	Polymer	Solvents			Additive	Viscosity
	PVDF (wt%)	DMSO (v/v)	DMF (v/v)	Acetone (v/v)	LiCl (wt%)	(cP)
DS1	8	-	6	4	0.43	87.3
DS2	8	6	-	4	-	79.4
DS3	6	6	-	4	0.43	49.6
DS4	8	6	-	4	0.43	74.4
DS5	10	6	-	4	0.43	199.3

**Table 3:** Hansen solubility parameters (HSPs) for polymer and solvents used in this work.

Compound	HSPs				Dielectric Constant [48]
	ẟ_T_ (MPa)	ẟ_d_ (MPa)	ẟ_p_ (MPa)	ẟ_h_ (MPa)	
PVDF [49]	24.2	17.2	12.5	9.2	-
DMF [49]	24.9	17.4	13.7	11.3	37.3
DMSO [49]	26.7	18.4	16.4	10.2	46.7

**Table 4:** Contact angle, porosity, pore size, and thickness of the prepared ENMs.

Code	Contact Angle (°)	Porosity (%)	Pore Size (µm)	Thickness (µm)
M1	125 ± 2	93 ± 1	0.81 ± 0.04	69 ± 1
M2	123 ± 3	74 ± 1	2.29 ± 0.03	49 ± 2
M3	128 ± 1	95 ± 1	2.26 ± 0.08	35 ± 1
M4	125 ± 3	92 ± 1	0.87 ± 0.08	65 ± 3
M5	125 ± 2	93 ± 1	0.90 ± 0.08	73 ± 1
M6	122 ± 2	97 ± 1	1.03 ± 0.02	76 ± 2
M7	127 ± 4	91 ± 3	1.27 ± 0.02	54 ± 2
M8	126 ± 1	95 ± 1	1.19 ± 0.04	67 ± 1
M9	128 ± 1	96 ± 1	1.06 ± 0.05	79 ± 1
M10	125 ± 2	96 ± 1	0.80 ± 0.02	42 ± 2
M11	114 ± 3	94 ± 1	0.93 ± 0.03	75 ± 1

**Table 5:** Most relevant works of PVDF ENMs membranes with different solvents.

Solvent ^1^	Toxicity ^2^	PVDF Conc. (wt.%)	Electrosp. Temp. (°C)	Fiber Appearance	Pore Size	Post Treatment	Ref.
DMA	Hazardous	12–16	Room temperature	Uniform PVDF nanofibers	2–5.88	-	[57]
		10–15	Continuous hot-press-2 layers	Some beads are present at lower concentrations	0.26–1.49	Dried at 50 °C for 24 h in the oven	[54]
DMF	Hazardous	19	Continuous hot-press at 145 °C;	Networks with large pores	-	40-55 °C for 12 h in the oven	[58]
		6	Room temperature	Some beads are present	0.91–1.12	Pressed between two flat glass panes and placed at 170 °C in an oven	[29]
NMP	Hazardous	16–20	Room temperature	Beads disappear and there are uniform fibres at lower concentration	-	100 °C in the oven	[59]
DMSO	Less Hazardous	6–10	Room temperature	Uniform nanofibers	0.8–2.29	Air for 2 h; water for 24 h and at 40 °C in the oven	This work
		15	Room temperature	Some beads are presents	-	-	[60]
		12	Room temperature	Uniform nanofiber	2.4	Heat treatment	[61]

^1^ In a mixture with acetone. ^2^ Regulation (EC) No 1272/2008

## References

[1] Figoli A., Simone S., Drioli E., Hilal N., Ismail A.F., Wright C. (2015). Polymeric membranes. Membrane Fabrication.

[2] Fane A.G., Tang C.Y., Wang R., Wilderer P. (2011). Membrane Technology for Water: Microfiltration, Ultrafiltration, Nanofiltration, and Reverse Osmosis. Treatise on Water Science.

[3] Liao Y., Loh C.-H., Tian M., Wang R., Fane A.G. (2018). Progress in electrospun polymeric nanofibrous membranes for water treatment: Fabrication, modification and applications. Prog. Polym. Sci..

[4] Haider A., Haider S., Kang I. (2015). A comprehensive review summarizing the effect of electrospinning parameters and potential applications of nanofibers in biomedical and biotechnology. Arab. J. Chem..

[5] Bhardwaj N., Kundu S.C. (2010). Electrospinning: A fascinating fiber fabrication technique. Biotechnol. Adv..

[6] Lv D., Zhu M., Jiang Z., Jiang S., Zhang Q., Xiong R., Huang C. (2018). Green Electrospun Nanofibers and Their Application in Air Filtration. Macromol. Mater. Eng..

[7] Cao J., Cheng Z., Kang L., Chu M., Wu D., Li M., Xie S. (2017). Novel stellate poly (vinylidene fluoride)/polyethersulfone microsphere- nanofiber electrospun membrane with special wettability for oil/water separation. Mater. Lett..

[8] Li K., Hou D., Fu C., Wang K., Wang J. (2019). Fabrication of PVDF nanofibrous hydrophobic composite membranes reinforced with fabric substrates via electrospinning for membrane distillation desalination. J. Environ. Sci..

[9] Su Q., Zhang J., Zhang L. (2020). Fouling resistance improvement with a new superhydrophobic electrospun PVDF membrane for seawater desalination. Desalination.

[10] Guillen G.R., Pan Y., Li M., Hoek E.M.V. (2011). Preparation and characterization of membranes formed by nonsolvent induced phase separation: A review. Ind. Eng. Chem. Res..

[11] (2020). ECHA, Candidate List of Substances of very High Concern for Authorisation, European Chemicals Agency. https://echa.europa.eu/.

[12] Marino T., Galiano F., Simone S., Figoli A. (2019). DMSO EVOL^TM^ as novel non-toxic solvent for polyethersulfone membrane preparation. Environ. Sci. Pollut. Res..

[13] Kerton F.M., Clark J.H., Kraus G.A. (2009). Alternative Solvents for Green Chemistry.

[14] Figoli A., Marino T., Simone S., Di Nicolò E., Li X.M., He T., Tornaghi S., Drioli E. (2014). Towards non-toxic solvents for membrane preparation: A review. Green Chem..

[15] Evenepoel N., Wen S., Tsehaye M.T., Bruggen B. (2018). Van Der Potential of DMSO as greener solvent for PES ultra- and nanofiltration membrane preparation. J. Appl. Polym. Sci..

[16] Dimethyl Sulfoxide, Safety Data Sheet, Sigma Aldrich. www.sigmaaldrich.com.

[17] Madaeni S.S., Bakhtiari L. (2012). Thermodynamic-based predictions of membrane morphology in water/dimethylsulfoxide/polyethersulfone systems. Polymer.

[18] Alexowsky C., Bojarska M., Ulbricht M. (2019). Porous poly (vinylidene fluoride) membranes with tailored properties by fast and scalable non-solvent vapor induced phase separation. J. Memb. Sci..

[19] Meringolo C., Mastropietro T.F., Poerio T., Fontananova E., De Filpo G., Curcio E., Di Profio G. (2018). Tailoring PVDF Membranes Surface Topography and Hydrophobicity by a Sustainable Two-Steps Phase Separation Process. ACS Sustain. Chem. Eng..

[20] Lin D.J., Chang C.L., Lee C.K., Cheng L.P. (2006). Preparation and characterization of microporous PVDF/PMMA composite membranes by phase inversion in water/DMSO solutions. Eur. Polym. J..

[21] Enayatzadeh M., Mohammadi T. (2018). Morphology and performance of poly (vinylidene fluoride) flat sheet membranes: Thermodynamic and kinetic aspects. J. Appl. Polym. Sci..

[22] Arefi-Oskoui S., Khataee A., Vatanpour V. (2017). Effect of solvent type on the physicochemical properties and performance of NLDH/PVDF nanocomposite ultrafiltration membranes. Sep. Purif. Technol..

[23] Wehlage D., Böttjer R., Grothe T., Ehrmann A. (2018). Electrospinning water-soluble/insoluble polymer blends. AIMS Mater. Sci..

[24] Sabantina L., Klöcker M., Wortmann M., Mirasol J.R., Cordero T., Moritzer E., Finsterbusch K., Ehrmann A. (2019). Stabilization of polyacrylonitrile nanofiber mats obtained by needleless electrospinning using dimethyl sulfoxide as solvent. J. Ind. Text..

[25] Gupta D., Jassal M., Agrawal A.K. (2016). The electrospinning behavior of poly(vinyl alcohol) in DMSO-water binary solvent mixtures. RSC Adv..

[26] Rianjanu A., Kusumaatmaja A., Suyono E.A., Triyana K. (2018). Solvent vapor treatment improves mechanical strength of electrospun polyvinyl alcohol nanofibers. Heliyon.

[27] Mondal S. (2014). Influence of solvents properties on morphology of electrospun polyurethane nanofiber mats. Polym. Adv. Technol..

[28] Wortmann M., Frese N., Sabantina L., Petkau R., Kinzel F., Gölzhäuser A., Moritzer E., Hüsgen B., Ehrmann A. (2019). New polymers for needleless electrospinning from low-toxic solvents. Nanomaterials.

[29] Liao Y., Wang R., Tian M., Qiu C., Fane A.G. (2013). Fabrication of polyvinylidene fluoride (PVDF) nanofiber membranes by electro-spinning for direct contact membrane distillation. J. Memb. Sci..

[30] Essalhi M., Khayet M. (2014). Self-sustained webs of polyvinylidene fluoride electrospun nano-fibers: Effects of polymer concentration and desalination by direct contact membrane distillation. J. Membr. Sci..

[31] Feng C., Khulbe K.C., Tabe S. (2012). Volatile organic compound removal by membrane gas stripping using electro-spun nanofiber membrane. Desalination.

[32] Liu C., Li X., Liu T., Liu Z., Li N., Zhang Y., Xiao C., Feng X. (2016). Microporous CA/PVDF membranes based on electrospun nanofibers with controlled crosslinking induced by solvent vapor. J. Membr. Sci..

[33] Kang G.D., Cao Y.M. (2014). Application and modification of poly(vinylidene fluoride) (PVDF) membranes—A review. J. Membr. Sci..

[34] Santoro S., Vidorreta I.M., Sebastian V., Moro A., Coelhoso I.M., Portugal C.A.M., Lima J.C., Desiderio G., Lombardo G., Drioli E. (2017). A non-invasive optical method for mapping temperature polarization in direct contact membrane distillation. J. Memb. Sci..

[35] Santoro S., Vidorreta I., Coelhoso I., Lima J.C., Desiderio G., Lombardo G., Drioli E., Mallada R., Crespo J., Criscuoli A. (2019). Experimental evaluation of the thermal polarization in direct contact membrane distillation using electrospun nanofiber membranes doped with molecular probes. Molecules.

[36] Deitzel J., Kleinmeyer J., Harris D., Beck Tan N. (2001). The effect of processing variables on the morphology of electrospun nanofibers and textiles. Polymer.

[37] Zhao Q., Xie R., Luo F., Faraj Y., Liu Z., Ju X.J., Wang W., Chu L.Y. (2018). Preparation of high strength poly(vinylidene fluoride) porous membranes with cellular structure via vapor-induced phase separation. J. Memb. Sci..

[38] Tarus B., Fadel N., Al-Oufy A., El-Messiry M. (2016). Effect of polymer concentration on the morphology and mechanical characteristics of electrospun cellulose acetate and poly (vinyl chloride) nanofiber mats. Alex. Eng. J..

[39] Mit-uppatham C., Nithitanakul M., Supaphol P. (2004). Ultrafine Electrospun Polyamide-6 Fibers: Effect of Solution Conditions on Morphology and Average Fiber Diameter. Macromol. Chem. Phys..

[40] Cengiz F., Jirsak O. (2009). The effect of salt on the roller electrospinning of polyurethane nanofibers. Fibers Polym..

[41] Qin X.-H., Yang E.-L., Li N., Wang S.-Y. (2007). Effect of different salts on electrospinning of polyacrylonitrile (PAN) polymer solution. J. Appl. Polym. Sci..

[42] Shirazi M.J.A., Bazgir S., Shirazi M.M.A., Ramakrishna S. (2013). Coalescing filtration of oily wastewaters: Characterization and application of thermal treated, electrospun polystyrene filters. Desalin. Water Treat..

[43] Son W.K., Youk J.H., Lee T.S., Park W.H. (2004). The effects of solution properties and polyelectrolyte on electrospinning of ultrafine poly (ethylene oxide) fibers. Polymer.

[44] Massaglia G., Quaglio M. (2018). Semiconducting nanofibers in photoelectrochemistry. Mater. Sci. Semicond. Process..

[45] Zhao Z., Li J., Yuan X., Li X., Zhang Y., Sheng J. (2005). Preparation and properties of electrospun poly (vinylidene fluoride) membranes. J. Appl. Polym. Sci..

[46] Burger C., Hsiao B.S., Chu B. (2006). Nanofibrous materials and their applications. Annu. Rev. Mater. Res..

[47] Hansen C.M. (2000). Hansen Solubility Parameters: A User’s Handbook. Choice Rev. Online.

[48] Smallwood I.M. (2002). Solvent Recovery Handbook.

[49] Russo F., Galiano F., Pedace F., Aricò F., Figoli A. (2020). Dimethyl Isosorbide As a Green Solvent for Sustainable Ultrafiltration and Microfiltration Membrane Preparation. ACS Sustain. Chem. Eng..

[50] Luo C.J., Stride E., Edirisinghe M. (2012). Mapping the Influence of Solubility and Dielectric Constant on Electrospinning Polycaprolactone Solutions. Macromolecules.

[51] Zhu X., Cui W., Li X., Jin Y. (2008). Electrospun Fibrous Mats with High Porosity as Potential Scaffolds for Skin Tissue Engineering. Biomacromolecules.

[52] Marino T., Russo F., Figoli A. (2018). The formation of polyvinylidene fluoride membranes with tailored properties via vapour/non-solvent induced phase separation. Membranes.

[53] Iulianelli A., Russo F., Galiano F., Desiderio G., Basile A., Figoli A. (2019). PLA Easy Fil—White-based membranes for CO_2_ separation. Greenh. Gases Sci. Technol..

[54] Lalia B.S., Guillen-Burrieza E., Arafat H.A., Hashaikeh R. (2013). Fabrication and characterization of polyvinylidenefluoride-co-hexafluoropropylene (PVDF-HFP) electrospun membranes for direct contact membrane distillation. J. Memb. Sci..

[55] Yang S., Wang X., Ding B., Yu J., Qian J., Sun G. (2011). Controllable fabrication of soap-bubble-like structured polyacrylic acid nano-nets via electro-netting. Nanoscale.

[56] Senthil T., Anandhan S. (2017). Effect of Solvents on the Solution Electrospinning of Poly (styrene-*co*-acrylonitrile). Testing and Measuring. www.kgk-rubberpoint.de.

[57] Akduman Ç. (2019). PVDF Electrospun Nanofiber Membranes for Microfiltration: The Effect of Pore Size and Thickness on Membrane Performance. Avrupa Bilim ve Teknoloji Dergisi.

[58] Na H., Zhao Y., Zhao C., Zhao C., Yuan X. (2008). Effect of hot-press on electrospun poly (vinylidene fluoride) membranes. Polym. Eng. Sci..

[59] Lei T., Zhan Z., Zuo W., Cheng W., Xu B., Su Y., Sun D. (2012). Electrospinning PVDF/EC fibre from a binary solvent system. Int. J. Nanomanuf..

[60] Saghafi H., Brugo T., Minak G., Zucchelli A. (2015). The effect of PVDF nanofibers on mode-I fracture toughness of composite materials. Compos. Part B Eng..

[61] Gee S., Johnson B., Smith A.L. (2018). Optimizing electrospinning parameters for piezoelectric PVDF nanofiber membranes. J. Membr. Sci..

